# Trapped Lung and Pneumothorax Ex Vacuo

**DOI:** 10.1002/ccr3.72083

**Published:** 2026-03-01

**Authors:** Mashal Binte Ali, Danish Jilani, Meher Binte Ali

**Affiliations:** ^1^ MountainView Regional Medical Center New Mexico USA; ^2^ University of Maryland Medical Center Baltimore Maryland USA

**Keywords:** hydropneumothorax, pleural effusion, pneumothorax, trapped lung

## Abstract

Trapped lung and pneumothorax ex vacuo occur due to failure of lungs to re‐expand after drainage of a large pleural effusion. Asymptomatic patients can be observed while symptomatic patients may need surgical decortication. Iatrogenic pneumothorax results from introduction of air into the pleural space and usually requires chest tube placement.

A 62‐year‐old male with a past medical history of metastatic adenocarcinoma of the lung complicated by recurrent malignant pleural effusion, emphysema, pulmonary fibrosis, and pulmonary hypertension presented with acute on chronic hypoxic respiratory failure. He had an indwelling left‐sided pleural catheter, from which he drained fluid three times a week. He required 4 L of oxygen at home. Due to hypoxia, he was placed on high‐flow nasal cannula. Chest x‐ray showed a large left‐sided pleural effusion, widespread coarsening of the interstitium, and emphysematous changes throughout both lungs (Figure 1). Almost half a liter of pleural fluid was drained daily for two days, and he was started on antibiotics for empyema due to gram‐positive cocci in the pleural fluid. On the third day, his pleural catheter was connected to a water seal, and he was able to be weaned down to his home oxygen requirements. A repeat x‐ray showed interval development of an area of trapped lung, pneumothorax ex vacuo, and hydropneumothorax (Figure 2). Repeated x‐rays showed stable size of trapped lung, and as he remained asymptomatic with no oxygen requirement beyond baseline, no surgical intervention was pursued.

**FIGURE 1:**
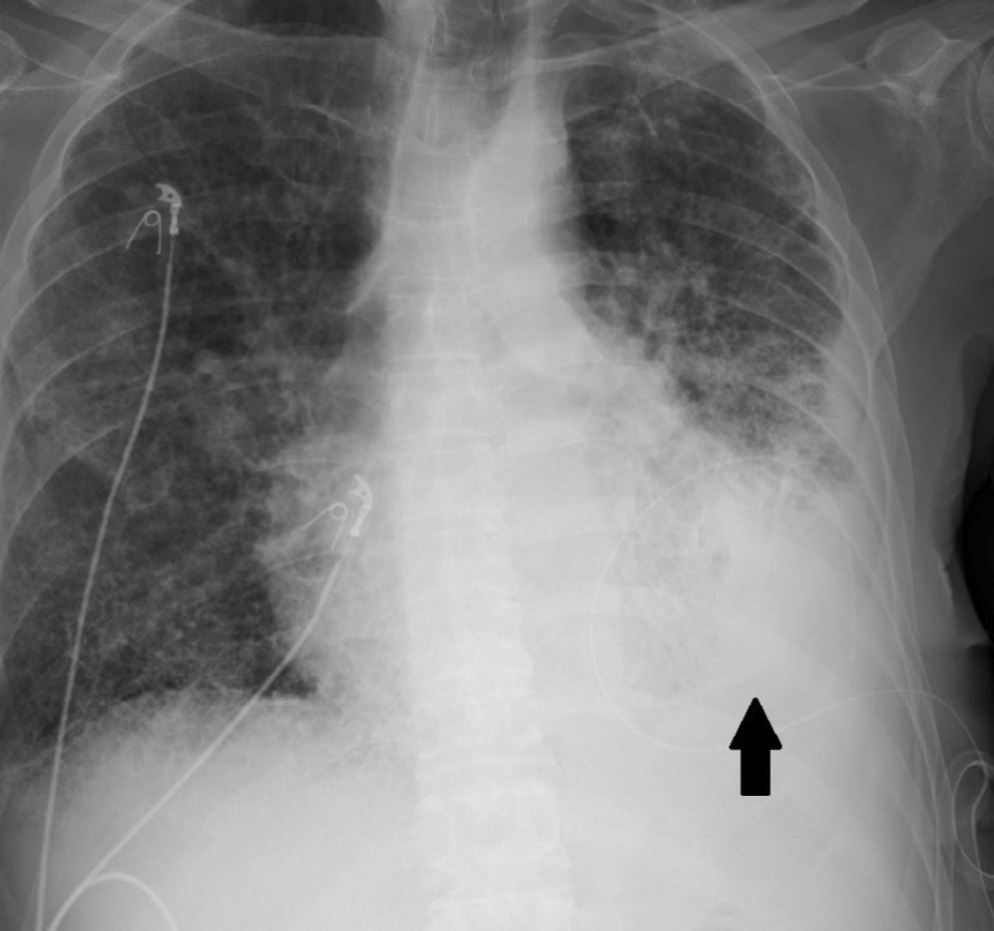
Chest x‐ray showing a large left‐sided pleural effusion.

**FIGURE 2:**
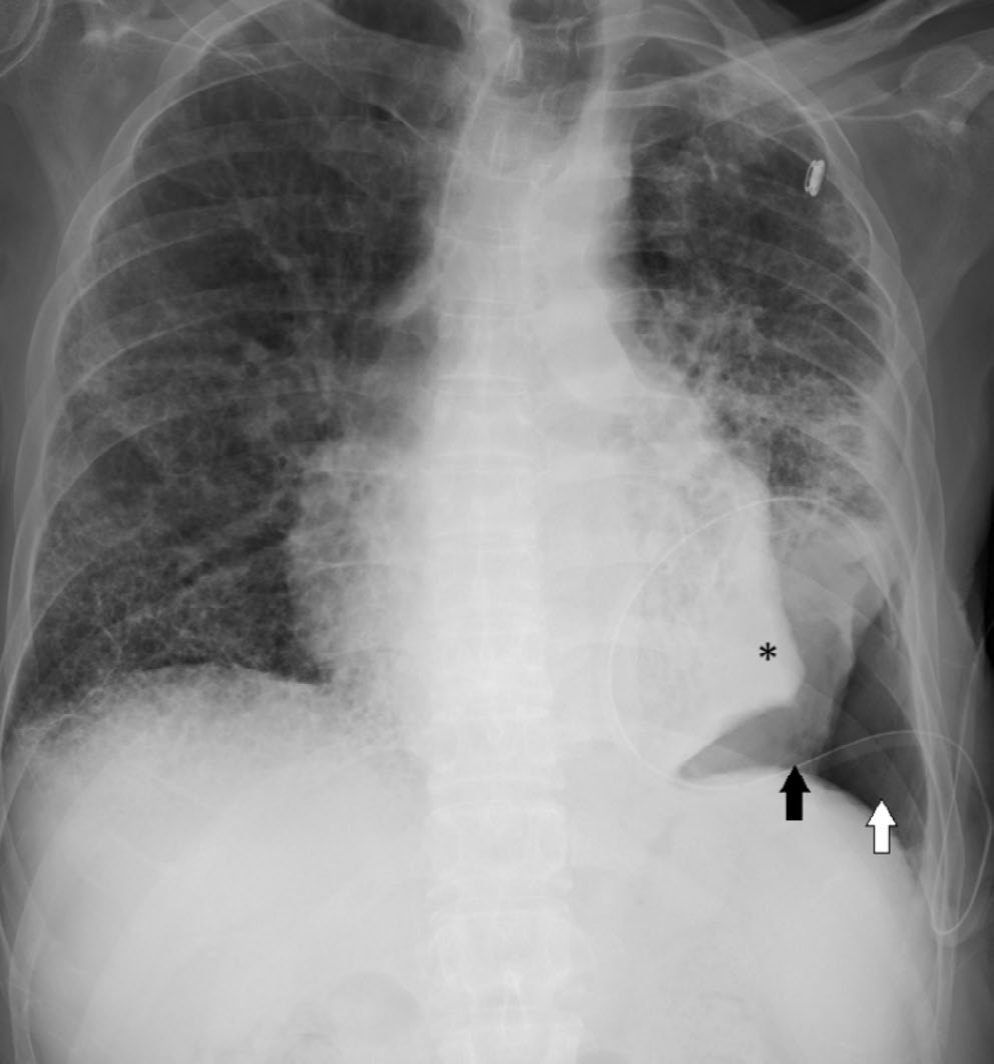
Chest x‐ray showing trapped lung (asterisk), pneumothorax ex vacuo (white arrow) and hydropneumothorax (black arrow).

Trapped lung occurs when the lung fails to fully re‐expand after pleural drainage due to the formation of a fibrous peel on the visceral pleura. This fibrous layer prevents normal apposition of the lung to the chest wall and is commonly associated with chronic inflammation from prior pneumonia, empyema, hemothorax, or malignancy [1]. A negative pressure gradient develops which draws air into the pleural space, causing a condition called pneumothorax ex vacuo. In contrast, iatrogenic pneumothorax typically results from direct pleural injury during procedures such as thoracentesis, central line placement, mechanical ventilation, or lung biopsy, which can introduce air into the pleural space [2]. It often requires chest tube drainage depending on size and symptoms. Both are important to distinguish as additional chest tubes in pneumothorax ex vacuo do not lead to re‐expansion of the lungs and can further worsen it.

Asymptomatic patients can be observed while symptomatic patients may benefit from surgical decortication to restore lung expansion. If the lung remains unexpandable, the pleural space may partially refill with fluid over time, leading to a hydropneumothorax. Most patients can be managed conservatively as long as they are clinically stable. In patients with underlying malignancy or chronic pleural disease, this condition often reflects advanced disease, and treatment goals should focus on symptom management and quality of life rather than aggressive interventions.

## Author Contributions

**Mashal Binte Ali:** writing – original draft, writing – review and editing. **Danish Jilani:** writing – original draft, writing – review and editing. **Meher Binte Ali:** conceptualization, investigation, writing – original draft, writing – review and editing.

## Funding

The authors have nothing to report.

## Consent

Written informed consent was obtained from the patient for publication of this case report and any accompanying images.

## Conflicts of Interest

The authors declare no conflicts of interest.

## Data Availability

Data sharing is not applicable to this article as no datasets were generated or analyzed during the current study.
